# Complete root coverage in the treatment of Miller class III or RT2 gingival recessions: a systematic review and meta-analysis

**DOI:** 10.1186/s12903-021-01494-3

**Published:** 2021-03-22

**Authors:** Aitziber Fernández-Jiménez, Ana-María García-De-La-Fuente, Ruth Estefanía-Fresco, Xabier Marichalar-Mendia, José-Manuel Aguirre-Urizar, Luis-Antonio Aguirre-Zorzano

**Affiliations:** 1 Department of Stomatology II, University of the Basque Country (UPV/EHU), UPV/EHU. Barrio Sarriena S/N, 48940 Leioa, Biscay Spain; 2 Department of Nursing I, University of the Basque Country (UPV/EHU), Barrio Sarriena S/N, 48940 Leioa, Biscay Spain

**Keywords:** Class III gingival recession, RT2 gingival recession, Plastic surgery, Treatment outcome

## Abstract

**Background:**

The primary objective of this systematic review and meta-analysis was to assess the evidence on complete root coverage (CRC) achieved by periodontal plastic techniques in the treatment of Miller class III/RT2 gingival recessions, comparing techniques developed along the twentieth century (pre-twenty-first) versus surgical approaches of the twenty-first century (21st).

**Methods:**

An electronic bibliographic search was carried out in four databases up to December 2019, focusing on studies that reported CRC results in Miller class III or RT2 recessions treatment with at least a six-month follow-up. In addition, a random-effects models’ meta-analysis was performed for the CRC, comparing pre-twenty-first versus twenty-first century techniques at 6 months, 12 months and more than 12 months.

**Results:**

Thirty-seven publications were included. A total of 933 gingival recessions were treated, 298 with pre-twenty-first century surgical techniques and 635 with techniques from the twenty-first century. CRC was achieved at 6 months on half of the recessions (pre-twenty-first: 57.60% vs. 21st: 51.11%), but decreased markedly for twenty-first century techniques at 12 months (pre-twenty-first: 63.82% vs. 21st: 32.87%). Thereafter, this difference was the other way around (> 12 months: pre-twenty-first: 5.26% vs. 21st: 19.65%). The meta-analysis showed a high heterogeneity, with no significant differences amongst the techniques.

**Conclusions:**

Although CRC might be achievable by treating Miller class III or RT2 recessions with any of the described techniques, its long-term stability is not predictable. More randomized clinical trials with longer follow-ups and several visits, are needed. In addition, the patient’s satisfaction should also be assessed.

**Supplementary Information:**

The online version contains supplementary material available at 10.1186/s12903-021-01494-3.

## Introduction

Periodontitis is a very prevalent pathology that ultimately leads to tooth loss in adult population [1–3]. As it progresses, multiple signs and symptoms may appear, including gingival recessions as a consequence of periodontal attachment loss. Most of the recessions in periodontal patients involve the destruction of interproximal periodontal tissues, therefore, they could be classified as Miller class III [4] or RT2 [5] gingival recessions (GRs).

Miller classification [4] has been the most commonly used for identifying the type of recession and for predicting the results of its treatment in terms of root coverage (RC). Nevertheless, with the development of new treatment options, this classification no longer matches the treatment outcomes expected to be achieved [6, 7]. In an attempt to overcome this limitation, a new classification based on the interdental clinical attachment level was proposed [5] which has been accepted by the American Academy of Periodontology [8] and the European Federation of Periodontology [9].

In order to treat these challenging Miller class III [4] or RT2 [5] recessions, several mucogingival approaches have been proposed such as tunnel techniques, coronally advanced flaps, free gingival grafts, rotated techniques and two-stage procedures. These techniques have been developed along the twentieth century (pre-twenty-first century techniques) and the twenty-first century as well. Although modern modifications of classical techniques have been proposed in this century, aiming to increase the blood supply in the recipient area, there is no evidence about their predictability.

The main objective of these different treatment options is achieving complete root coverage (CRC), which has been accepted as the best indicator of success [10, 11]. However, when considering the effectiveness of these techniques, it would be important to assess the percentage of RC [10] since a mean defect coverage of 80%-100% could also be a successful outcome [12].

Taking all this into account, a systematic review was carried out in order to collect the evidence about CRC outcomes achieved with techniques developed along twentieth century (pre-twenty-first century) versus more current surgical approaches (twenty-first century) used for the treatment of Class III [4] or RT2 [5] recessions.

## Methods

### Review design and registration

This systematic review was carried out following the Preferred Reporting Items for Systematic Review and Meta-Analysis (PRISMA) guidelines [13]. The protocol has previously been registered in the International Prospective Register of Ongoing Systematic Reviews (PROSPERO) in 2018 [CRD42018103599 Available from: http://www.crd.york.ac.uk/PROSPERO/display_record.php?ID=CRD42018103599].

### Review question and search strategy

The focused PICO question was: In patients with Class III [4] or RT2 [5] gingival recessions (population), what percentage of CRC (primary outcome) can we expect when using older (pre-twenty-first century) versus more modern (twenty-first century) mucogingival techniques?

A bibliographic search was performed in December 2019, in the National Library of Medicine (MEDLINE via PubMed), Web of Science, Cochrane Library and Scopus databases. The search strategy included the combination of the following keywords: “Class III recession”, “RT2 recession” and “treatment”: ((Class III recession) OR (RT2 recession)) AND (treatment).

It has been postulated that a follow-up of at least 6 months might be necessary in order to predict long-term outcomes of these procedures [14, 15], so only clinical studies with a follow-up at least of 6 months were included in this systematic review.

### Eligibility criteria

Articles were included in this systematic review if they met the following inclusion criteria: (1) clinical studies including randomized clinical trials, cohort studies, retrospective studies, cases series and case reports in humans; (2) treatment of Miller class III or RT2 GRs around teeth (3) CRC was reported or could be obtained from the results provided; (4) that the results reported had at least six-month follow-up; (5) studies written in English and Spanish.

Instead, articles were to be excluded if: (1) they treated Miller class III or RT2 GRs, but there was no information about CRC or it was not possible to obtain this parameter; (2) letters and abstracts of meetings; (3) the resolution of the GR was not due to mucogingival treatment.

### Data extraction and quality of studies

Screening of eligible studies, data extraction and risk of bias assessment (Grading of Recommendations. Assessment, Development and Evaluation (GRADE) system) [16] were performed independently by two different examiners (AFJ and AMGF), using the same inclusion and exclusion criteria. A third researcher (REF) was consulted in case of disagreements.

The year 2001 was taken as a reference point to divide the two study-groups. Pre-twenty-first century techniques included all surgical approaches (tunnel techniques, coronally advanced flaps, free gingival grafts, rotated techniques and two-stage procedures) developed between 1902 and 2000, and those developed from 1/01/2001 until now were considered as twenty-first century techniques.

The quality of each type of study was assessed individually (in each included study) and globally (calculating percentages of all included studies) using risk of bias tables (GRADE system) [16]. In these tables, the following six parameters were recorded: random sequence generation, allocation concealment, blinding of participants and personnel, lack of incomplete outcome data, lack of selective reporting and being free of source of funding.

### Strategy for data synthesis

The main objective was to assess the percentage of CRC (treatment success) obtained in the treatment of Miller class III [4]/RT2 [5] recessions comparing the mucogingival techniques described before twenty-first century (pre-twenty-first century) versus the more recently described or modified techniques (twenty-first century). In the studies in which other types of recessions were treated in addition to Miller class III [4] or RT2 [5] GRs, only the data corresponding to these types of recessions were collected.

Likewise, mean baseline gingival recession depth as well as changes in other parameters, such as probing depth (PD), clinical attachment level (CAL) and keratinized tissue width (KTW) were recorded in millimeters.

For all the meta-analysis conducted, a random-effects model analysis was used. Heterogeneity was evaluated with Cochran´s Q test and I2 statistic. The principal parameter was the percentage of CRC, which was used for consistency measurements comparing pre-twenty-first century versus twenty-first century techniques in each follow-up, at 6 months, 12 months and more than 12 months. In addition, a cumulative meta-analysis for each follow-up time was performed, regardless of the technique used, in order to observe if there was any trend over time. Statistical significance was set at p < 0.05.

## Results

### Study selection

**Fig. 1:**
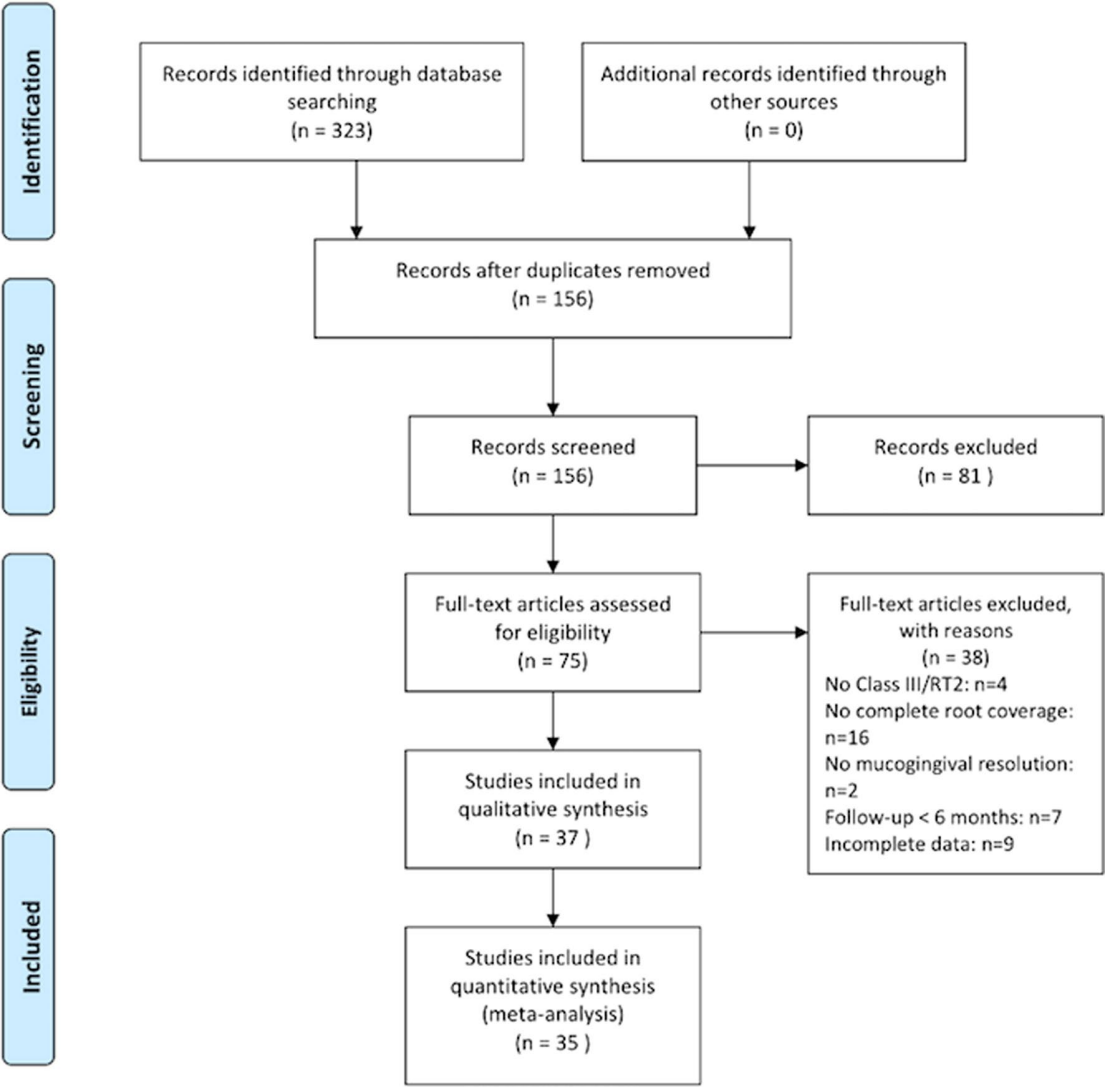
PRISMA Flow chart of the systematic review

The PRISMA flow chart in Fig. 1 summarizes the retrieval process of the studies included in this systematic review. A total of 323 studies were found, 134 in PubMed database, 143 in Web of Knowledge, 28 in The Cochrane Library and 18 in Scopus.

After excluding duplicates, 156 studies were screened. The titles and the abstracts of all reports identified were read separately by the two authors (AFJ and AMGF) to include the articles where Miller class III [4]/RT2 [5] were treated with periodontal plastic surgical procedures; the inter-examiner global agreement was of 95.68% (Kappa = 0.91). Articles with no sufficient information in their title or abstract to discard them were also included. Finally, 75 full-text articles were assessed for eligibility. Thirty-eight studies were excluded (Fig. 1) with a global agreement of 94.12%. Excluded studies and reasons for their exclusion are summarized on Additional file 1 (see Additional file 1).

Regarding the surgical techniques of GRs included in this systematic review, a total of 27 different surgical techniques were identified that were divided in two study groups: pre-twenty-first century [17–27] versus twenty-first century [6, 28–42] techniques (Table 1).

**Table 1:** Periodontal plastic surgical techniques used in Class III or RT2 recessions treatment

Pre-twenty-first century	twenty-first century
*Tunnel Technique (TT)*	
	Allen [34]
	Aroca et al. [6] (MCAT)
	Chao [36] (PST)
	Ribeiro et al. [31]
	Sculean and Allen [41](LCT)
	Tözüm and Dini [29]
	Zadeh [37] (VISTA)
*Coronally advanced flap (CAF)*	
Allen [23]	Zucchelli et al. [33](MCAF)
Allen and Miller [24]	Mercado et al. [42]
Ito et al. (PCTG) [26]	
Langer and Langer [20]	
Marggraf (BFP) [21]	
Zucchelli and De Sanctis [27]	
*Free gingival graft (FGG)*	
Holbrook and Ochsenbein [19]	Allen and Cohen [28] (GUT)
*Rotated Technique (ROT)*	
Grupe and Warren [17] (LPF)	Deliberador et al. [35] (LPF + TT)
Harris [25] (DPG)	Chambrone and Chambrone [32] (MLPF)
Nelson [22] (DPG)	Lee et al. [39] (MLPF)
	Zucchelli et al. [30] (LMCAF)
*Two-stage procedures (2SP)*	
Bernimoulin et al. [18] (FGG + CAF)	Núñez et al. [40] (2SSA: Odontoplasty/TT: Allen)
	Zucchelli and De Sanctis [38] (Modified 2SP: FGG + LMCAF)

MCAT: Modified coronally advanced tunnel; VISTA: Vestibular incision subperiosteal tunnel access; PST: Pinhole surgical technique; LCT: Laterally closed tunnel; BFP: Bridge flap procedure; PCTG: Periosteal connective tissue grafting; MCAF: Modified coronally advanced flap; GUT: Gingival unit transfer; DPG: Double pedicle graft; LMCAF: Laterally moved coronally advanced flap; MLPF: Modified laterally positioned flap; LPF: Laterally positioned flap; 2SSA: Two-Step Surgical Approach

### Study design and study population

Seventeen studies were cases series [39–41, 43–56], nine were case reports [31, 35, 38, 57–62], seven were clinical trials [6, 7, 14, 42, 63–65], and four were retrospective studies [36, 66–68]. Out of these 37 studies, only two studies [7, 65] used the classification proposed by Cairo et al. [5]. Almost half of the studies (n = 16) included only Miller class III [4] or RT2 [5] recessions while, in the rest (n = 18) Miller class I/II [4] or RT1 [5] or Miller class IV [4] (n = 1) recessions were also treated (Table 2).

**Table 2:** Characteristics of studies included for the systematic review and meta-analysis

Class III /RT2 studies (n = 37)	Study type	N RC Class III/RT2	RC location	Follow-up (months)	Surgical recipient site		Graft type
	(Class)				Technique	Author	
Case report (n = 9)							
Cizza and Migues [57]	III S	1	Mx 2.3	6/12	DPG‡	Harris [25]	SCTG
Deliberador et al. [35]	III S	1	Md 3.1	12	LPF + TT†	Deliberador et al. [35]	SCTG
Gajendran and Parthasarathy [58]	III M	2	Md 3.1, 4.1	12	GUT†	Allen and Cohen [28]	FGG
Luthra et al. [59]	III M	2	Mx 2.3/2.4	6/12	CAF‡	Allen [23]	Periosteal pedicle graft + Autogenous bone
Moussa and Bissada [60]	III M	6	Mx 1.5/1.3/1.2/ 1.1	12	TT†	Allen [34]	ADM
			Mx 2.4/ 2.6		MCAF†	Zucchelli et al. [33]	SCTG
Rath et al. [61]	III S	1	Md 4.1	6	2SP (FGG/CAF)‡	Bernimoulin et al. [18]	FGG/BB (BioMed®)
Rath et al. [62]	III S	1	Md 4.1	6	FGG‡	Holbrook and Ochsenbein [19]	FGG
Ribeiro et al. [31]	III S	1	Mx 2.4	36	TT†	Ribeiro et al. [31]	SCTG
Zuchelli and De Sanctis [38]	III S	1	Md 3.6	12/60	M2SP (FGG/LMCAF)†	Zuchelli and De Santics [38]	FGG/No
*Case series (n* = *17)*							
Boltchi et al. [43]	I, II,III S or M	14	NR	6	CAF‡	Allen [23]	BB (Guidor®)
Carnio et al. [44]	II, III S	2	Mx/Md 1.3/2.3	6/12	DPG‡	Nelson [22]	SCTG
Cosgarea et al. [45]	I, II, III M	25	Mx/Md incisors, canines or premolars	12	MCAT†	Aroca et al. [6]	ADM (Mucoderm®)
Garg et al. [46]	I and III M	9	Mx incisor, canines and premolar	6	VISTA†	Zadeh [37]	BB (PRF) /No
Gupta et al. [47]	I, II,III S or M	7	Md incisors	9	BFP‡	Marggraf [21]	No
Jepsen et al. [48]	I,II,III S	8	Mx/Md 1.3/ 2.3/ 3.3	12	CAF‡	Allen [23]	BB (Atrisorb®)
*Case series (n* = *17)*							
Lee et al. [39]	Only III S	2	Md 4.3	6/36	MLPF†	Lee et al. [39]	SCTG
Nart et al. [49]	II and III S or M	7	Md incisors	11.70 (6.21)	CAF‡	Zucchelli and De Sanctis [27]	SCTG
Nart and Valles [50]	II and III S	7	Md incisors	20.53 (8.89)	TT†	Tözüm and Dini [29]	SCTG
Núñez et al. [40]	II and III S	7	Md 3.1, 4.1	12	2SSA (Odontoplasty/TT)†	Núñez et al. [40]	SCTG
Pini Prato et al. [51]	I and III, S	25	Mx/Md incisors, canines or premolars	12/ 240	CAF‡	Allen and Miller [24]	No
Romanos et al. [52]	I, II and III M	48	Mx/Md	12	MCAT†	Aroca et al. [6]	ADM (Alloderm®)
Sato et al. [53]	Only III M	4	Md central incisors	12/24	PCTG‡	Ito et al. [26]	SCTG + EMD
Sculean et al. [54]	I, II and III M	5	Mx	12	MCAT†	Aroca et al. [6]	SCTG + EMD
Sculean et al. [55]	I and III S or M	7	Mx	12	MCAT†	Aroca et al. [6]	SCTG
Sculean and Allen [41]	I, II and III S	10	Md incisors and canines	12	LCT†	Sculean and Allen [41]	SCTG + EMD
Yaman et al. [56]	Only III M	68	Mx/Md	12	MCAT†	Aroca et al. [6]	SCTG
*Clinical trials (n* = *7)*							
Aroca et al. [6]	Only III M	139	Mx/Md	6/12	MCAT†	Aroca et al. [6]	SCTG/ + or—EMD
Cairo et al. [7]	Only RT2 S	29	Mx incisors, canines and premolars	6	CAF‡	Allen and Miller [24]	SCTG/ No
Cueva et al. [14]	I, II and III S or M	7	Mx/Md incisors, canines and premolars	6	CAF‡	Allen and Miller [24]	EMD/No
Mercado et al. [42]	III and IV M	127	Md incisors and canines	36	CAF†	Mercado et al. [42]	SCTG/ + or—EMD
Ozcelik et al. [63]	I, II and III S	30	Mx/ Md incisors and canines	6	MLPF†	Chambrone and Chambrone [32]	No
Ozcelik et al. [65]	RT1 and RT2 S	62	Mx/Md incisors and canines	6	CAF‡	Allen and Miller [24]	SCTG
Ucak et al. [64]	Only III S	50	Mx/Md incisors and canines	6	LMCAF†	Zucchelli et al. [30]	No
*Restrospective studies (n* = *4)*							
César-Neto et al. [66]	II and III S	3	Mx/Md incisors, canines and premolars	6	CAF‡	Langer and Langer [20]	SCTG
		4			DPG/LPF‡	Harris [25]/Grupe and Warren [17]	
Chao [36]	I, II and III S or M	36	Mx/Md	15 (5.2)	PST†	Chao [36]	BB (Bio-Gide®)/ADM (Alloderm®)
Esteibar et al. [67]	Only III, S or M	121	Mx/Md	12	FGG‡	Holbrook and Ochsenbein [19]	FGG
					CAF‡	Langer and Langer [20]	SCTG
					DPG‡	Harris [25]	
Gil et al. [68]	I, II and III M	54	Mx/Md	≥ 12 (14.6 (4.6))	VISTA†	Zadeh [37]	SCTG/ADM (Perioderm®) /XCM (Mucograft®) + PDGF (GEM21S®)

S: single; RC: Recession; M: multiple; Mx: Maxilla; Md: Mandible; NR: No reported; †: twenty-first century technique; ‡: pre-twenty-first century technique; GUT: Gingival unit transfer; CAF: Coronally avanced flap; FGG: Free gingival graft; TT: Tunnel technique; MCAF: Modified coronally advanced flap; 2SP: Two-stage procedures; 2SSA: Two-stage surgical approach; LMCAF: Laterally moved coronally advanced flap; DPG: Double pedicle graft; LPF: Laterally positioned flap; LCT: Laterally closed tunnel; VISTA: Vestibular incision subperiosteal tunnel access; MCAT: Modified coronally advanced tunnel; MLPF: Modified laterally positioned flap; BFP: Bridge flap procedure; PCTG: Periosteal connective tissue grafting; PST: Pinhole surgical technique; ADM: Acelullar dermal matrix; XCM: Xenogenic collagen matrix; SCTG: Subepithelial connective tissue graft; BB: Bioabsorbable barrier; EMD: Enamel matrix derivative; PRF: Platelet-rich fibrin; PDGF: Platelet derived growth factor

Of the 16 articles focusing only on Miller class III GRs, nine of them [7, 31, 35, 38, 39, 57, 61, 62, 64] treated single GRs, six studies [6, 53, 56, 58–60] included multiple GRs, and one study [67] included single or multiple Miller class III GRs. In the rest of articles, different kind of GRs were treated: single Miller class I, II and III GRs were treated in nine studies [40, 41, 44, 48, 50, 51, 63, 65, 66], whereas multiple Miller class I, II, III and IV GRs were treated in five articles [45, 46, 52, 54, 68], and single and multiple Miller class I, II and III GRs were treated in other seven articles [14, 42, 43, 46, 47, 49, 55] (Table 2).

### Type of intervention

In the treatment of Miller class III [4] or RT2 [5] recessions, the tunnel preparation and the CAFs were the most widely used surgical techniques. Regarding surgical procedures developed along twentieth century, they were used in 16 clinical studies [7, 14, 43, 44, 47–49, 51, 53, 57, 59, 61, 62, 65–67] (Table 2).

In the majority of the studies, a subepithelial connective tissue graft or free gingival graft was used, followed by other alternatives such as connective tissue substitutes (acellular dermal matrix, porcine acellular dermal matrix) [36, 45, 52, 60, 68], bioabsorbable membranes (collagen membranes, PRF membranes) [36, 43, 46, 48], platelet-derived growth factors (GEM21S®) [68] and enamel matrix proteins (Emdogain®) [6, 14, 42]. In 3 studies [47, 51, 63] no grafts were used (Table 2).

### Risk of bias assessment

**Fig. 2:**
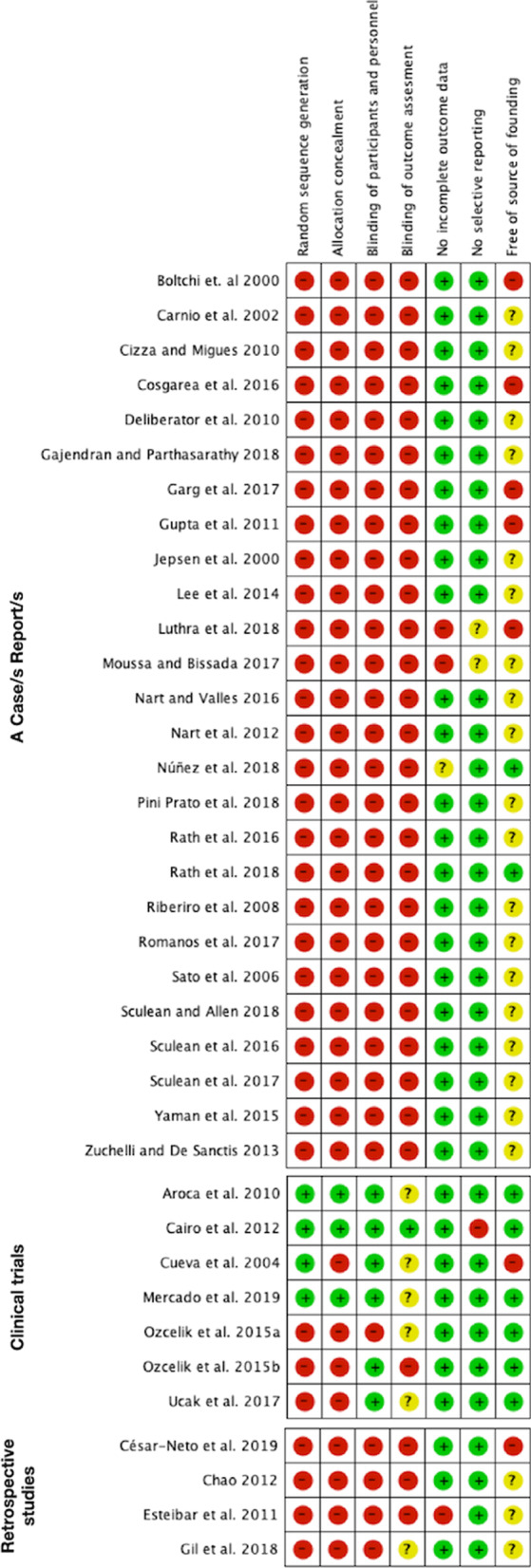
Summary of the risk of bias, assessing each risk of bias item in each included study

**Fig. 3:**
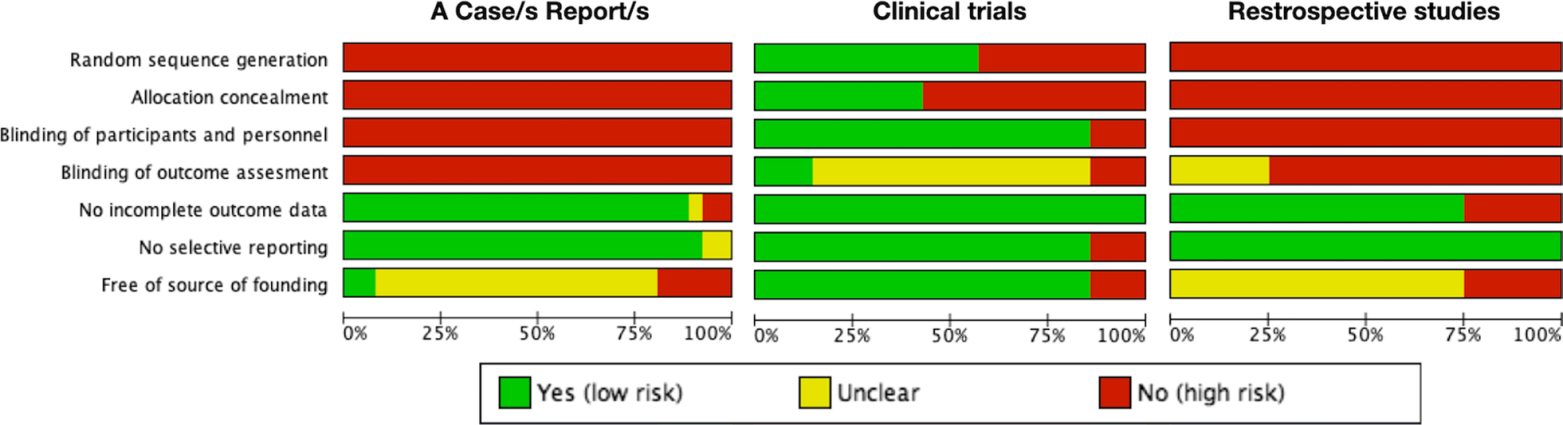
Graph of the risk of bias, presenting each risk of bias item as a percentage across all the included studies

Current evidence, mainly based on case reports and cases series, presented a high risk of bias (Fig. 2). Nevertheless, 7 clinical trials provided a higher level of evidence (Fig. 3).

### Synthesis of results

To quantitatively address the focused question of this review, data from articles were extracted and organized into tables to condense an overview of intervention characteristics and clinical outcomes. Articles in the tables were organized according to the type of study, as well.

A total of 933 Miller class III [4] or RT2 [5] GRs from 37 studies were evaluated in the present systematic review. Of these lesions, 298 were treated with pre-twenty-first century surgical techniques [17–27] and 635 GRs with techniques [6, 28–42] from the twenty-first century. They were mainly single recessions involving incisors, canines and premolars of both jaws (Table 2).

Baseline measurements of the RC, PD and KTW, as well as the number of recessions that showed CRC and the corresponding percentage of the total, are shown in Table 3. There were no clinical differences (mean < 1 mm) between the two treatment groups in baseline measurements of recession, PD and KTW. Although most studies had followed-up these recessions up to 6 and 12 months, only one clinical trial [42] and two cases studies [38, 51] reported results in advance, at three, five and twenty years, respectively.

**Table 3:** Clinical results of the studies included in the systematic review and meta-analysis

Class III/R T2 studies (n = 37)	No RC	Complete root coverage									Periodontal clinical parameters (mm)														
		6 Mo		9 Mo		12 Mo		> 12 Mo			RC t0	PD t0	PD change				CAL change (- gain)				KTW t0	KTW change			
		n	%	n	%	n	%	n	%	M			6 Mo	9 Mo	12Moo M	> 12 Mo	6 Mo	9 Mo	12 Mo	> 12 Mo		6 Mo	9 Mo	12 Mo	> 12 Mo
*A case report (n* = *9)*																									
Cizza and Migues [57] ‡	1	0	0			0	0				7	2	0		0		-5		-3.50		0.50	+ 4		+ 4	
Deliberador et al. [35]†	1					0	0				7	1			-0.50				-4.50						
Gajendran and Parthasarathy [58]†	2					2	100				3.50	2			-1				-5.50		1.50			+ 1.5	
Luthra et al. [59]‡	2	2	100			2	100																		
Moussa and Bissada [60]†	4					3	75				3.50	1.25									3.50			0	
Rath et al. [61]‡	2	NR			1	50					4.50	1									1.50			+ 1	
	1	0	0		NR						8	NR									1	+ 8			
Rath et al. [62]‡	1	1	100								7	2	0				-7				1	+ 7			
Ribeiro et al. [31]†	1							0	0	36	4														
Zuchelli and De Sanctis [38]†	1					0	0	0	0	60	6	4			-3	-3 (60 M)			-7	-7 (60 M)	0			+ 3	+ 3 (60 M)
*Case series (n* = *17)*																									
Boltchi et al. [43]‡	14	7	50																						
Carnio et al. [44]‡	1	0	0		NR					6	1	0		NR		-3		NR		1	+ 3		NR		
	1	NR			0	0				6	2	NR		0		NR		-4		0	NR		+ 5		
Cosgarea et al. [45]†	25					9	36																		
Garg et al. [46]†	9	2	40								2.80	3.48	-1.73				-3.45								
Gupta et al. [47]‡	7			0	0						3.57	1.64		-0.30				-1.85			1.50		+ 3.50		
Jepsen et al. [48]‡	8					0	0				5.40	2.30			-0.66				-4.49		1.94			+ 2.69	
Lee et al. [39]†	1	0	0					NR		36	9														
	1	NR						0	0		8														
Nart et al. [49]‡	7					3	42.85		5.14			1.71			-0.71				-5.01		0			+ 3	
Nart and Valles [50]†	7							1	14.30	20	4.57	1.14				0 (20 M)				-3.35 (20 M)	0				+ 2.57 (20 M)
Núñez et al. [40]†	7					2	28.50				6.07	1.29			-0.15				-4.64		0			+ 5.71	
Pini Prato et al. [51]‡	8					3	12	NR		240	2.68	1.12			-0.04	-0.12 (240 M)			-1.64	-0.94 (240 M)	3.40			-0.32	-0.77 (240 M)
	17				NR		0	0			NR				NR				NR		NR				
Romanos et al. [52]†	48					17	35.40																		
Sato et al. [53]‡	2					2	100	NR		24	3.50	1			+ 0.50	NR			-3	NR	0.75			+ 1.25	NR
	2					NR		1	50		3.50	1.50			NR	+ 0.50 (24 M)			NR	-2.50 (24 M)	0.25			NR	+ 3.75 (24 M)
Sculean et al. [54]†	5					1	20																		
Sculean et al. [55]†	7					6	85.70																		
Sculean and Allen [41]†	10					6	60																		
Yaman et al. [56]†	68					34	50				2.23														
RCTS (n = 7)																									
Aroca et al. [6]†	139 (40 pt)					16 pt	40				3.35	1.45	-0.30		-0.20		-3		-2,85		2.55	+ 0.2		+ 0.1	
Cairo et al. [7]‡	29	12	42.86								2.75	1.40	0				-2.15				2.70	+ 0.65			
Cueva et al. [14]‡	7	3	42.86																						
Mercado et al. [42]†	127					26	20.47	18	14.17	36	5.61	1.12			+ 0.18	+ 0.20 (36)			-4.05	-3.43 (36)	1.61			+ 1.72	+ 1.87 (36)
Ozcelik et al. [63]†	30	4	13.30								6.20	1.33	+ 0.27*				-0.42*				0	+ 4.47*			
Ozcelik et al. [65]‡	62	46	74.20								6	1.60	-0.10				-5.9				1.8	+ 5.7			
Ucak et al. [64]†	50	40	80								4.34	1.68	+ 0.08				-4.02				0.68	+ 4.12			
*Restrospective studies (n* = *4)*																									
César-Neto et al. [66]‡	3	0	0						5.83		0.33	+ 2.33													
	4	1	25						6.63		0	+ 4.13													
Chao [36]†	36							15	41.70	15	3.60	2.40				-1.10 (15)				-3.8 (15)	0				+ 2.5 (15)
Esteibar et al. [67]‡	11					1	11																		
	102					84	82																		
	8					1	7																		
Gil et al. [68]†	54					12	22.20				2.50	2.10			2.10				-2.10		1.80			+ 0.50	

pt, patient; NR, Not reported; Mo, Months; RC, Recession; PD, Probing depth; CAL, Clinical attachmente level; KTW, Keratinized tissue width

^†^Twenty-first century technique; ^†^Pre-twenty-first century technique;

### Complete root coverage

Regardless of the technique used in the treatment of Miller class III [4] or RT2 [5] recessions, CRC percentage of 54.88% (n = 118/215 GRs), 42.07% (n = 215/511 GRs) and 18.23% (n = 35/192 GRs) was reported in the 6-, 12- and more than 12-months follow-up points, respectively (Table 3).

Hence, breaking down this variable according to the technique used, a CRC percentage of 57.60% (n = 72/125 GRs) and 51.11% (n = 46/90 GRs) was observed at six months of 63.82%, (n = 97/152 GRs) and 32.87% (n = 118/359 GRs) at 12 months and decreasing beyond 12 months, to a 5.26% (n = 1/19 recessions) and 19.65% (n = 34/173 GRs), for the pre-twenty-first and twenty-first century treatment techniques, respectively.

### Periodontal parameters (PD, CAL, KTW)

Changes in some of the clinical parameters (PD, CAL, and KTW) were reported in 25 studies (Table 3**).** Regarding PD, an average reduction of less than 1 mm was observed in both groups, in the three time-points (six, 12 and beyond 12 months).

In relation to the CAL, at six-month follow-up, a higher gain was observed for the pre-twenty-first century treatment group (pre-twenty-first: 4.61 mm vs. 21st: 2.72 mm). However, the opposite was seen in the 12-month (pre-twenty-first: 3.61 mm vs. 21st: 4.38 mm) and in the beyond 12-month (pre-twenty-first: 1.72 mm vs. 21st: 4.40 mm) evaluations.

Finally, concerning the KTW, a significant gain could be observed for both treatment groups at six-month follow-up (pre-twenty-first: 4.35 mm vs. 21st: 2.93 mm), which was reduced at the 12-month assessment (pre-twenty-first: 2.37 mm vs. 21st: 1.79 mm). However, after 12 months of follow-up, the gain in the KTW seemed to remain stable around 2 mm in both groups (pre-twenty-first: 1.49 mm vs. 21st: 2.48 mm). Pini-Prato et al. [51] observed a reduction in the KTW from 12 months on (12 months: − 0.32 mm/240 months: − 0.77 mm).

### Meta-analysis

**Fig. 4:**
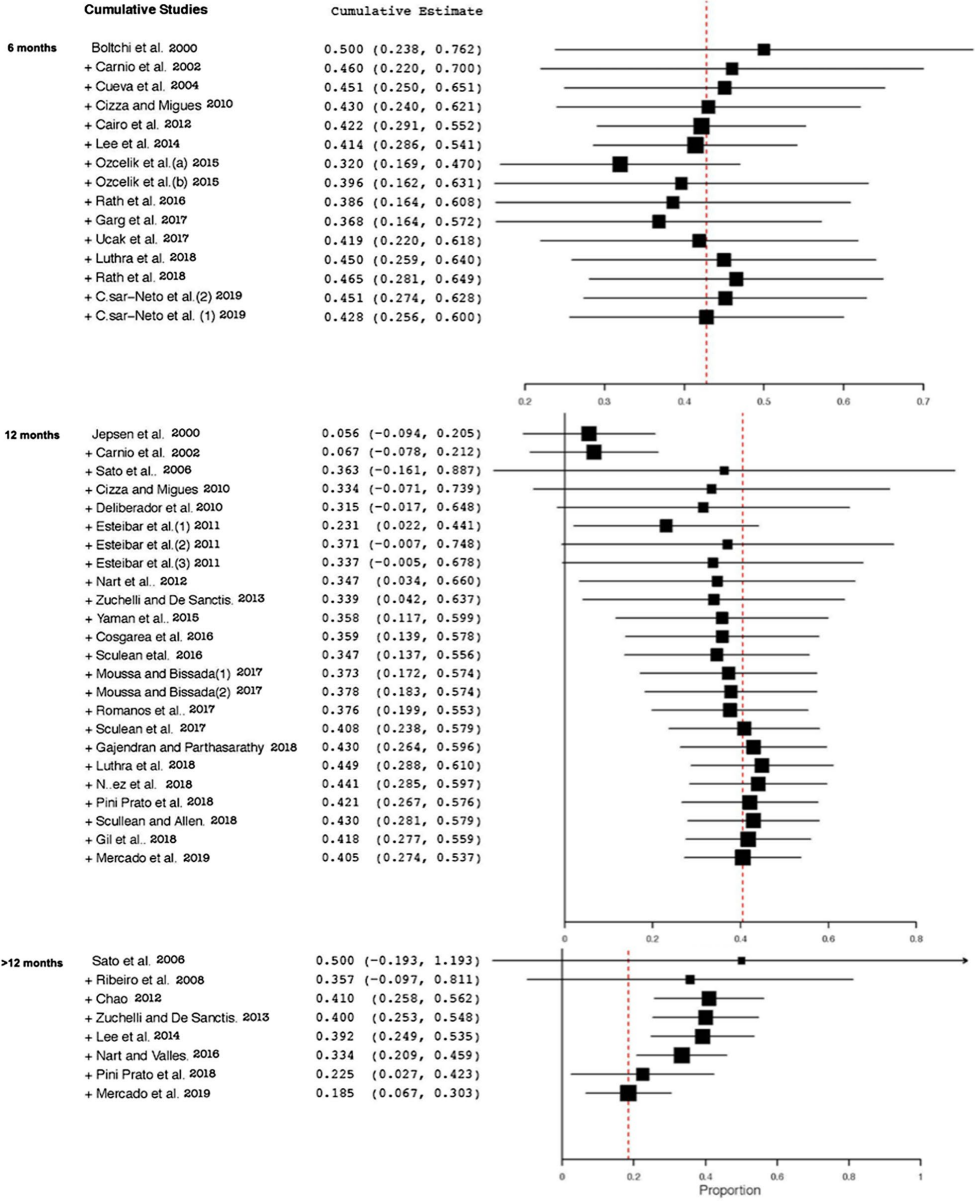
Random-effects model analyses comparing pre-twenty-first century and twenty-first century techniques

**Fig. 5:**
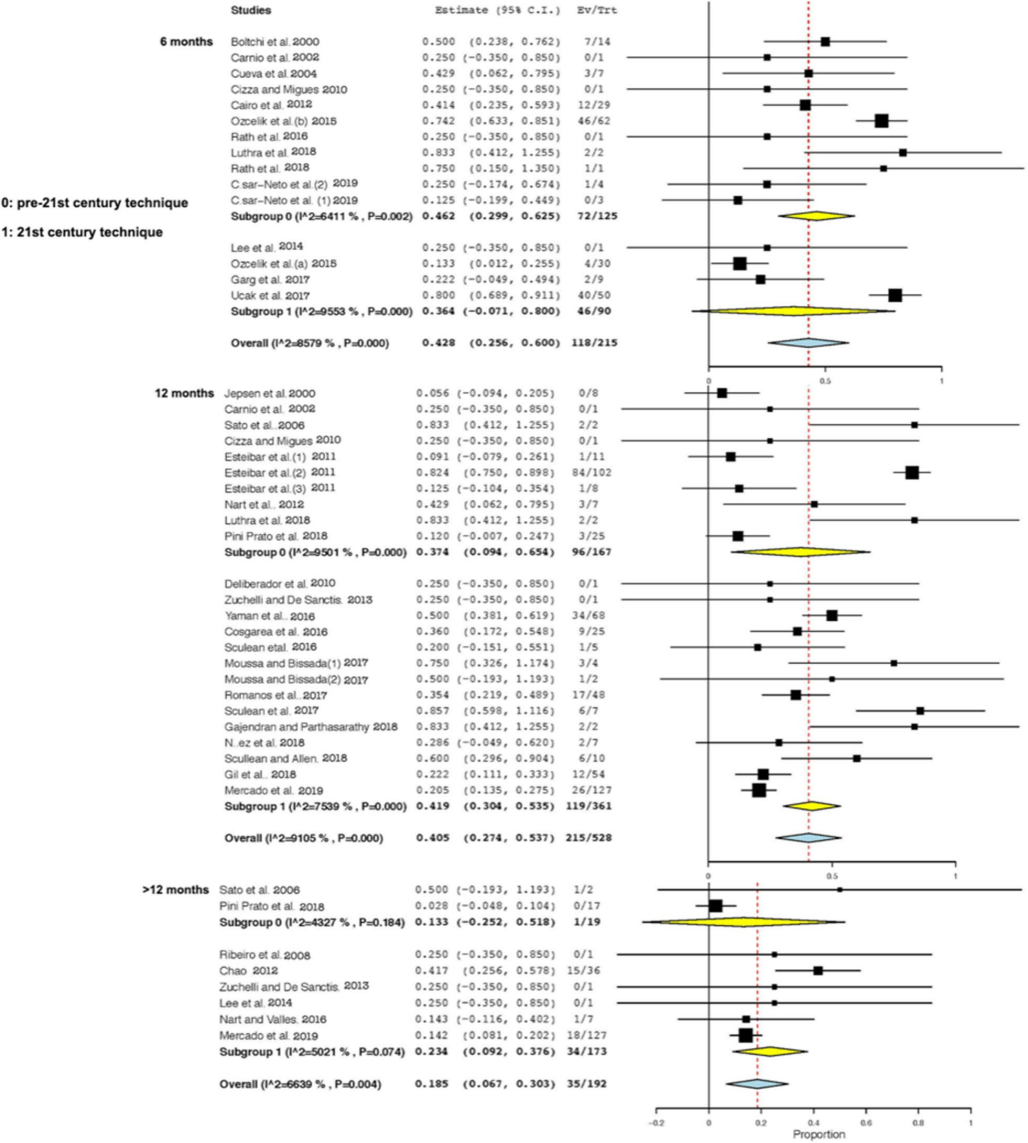
Cumulative meta-analysis for all studies

Although a total of 37 studies were included for the qualitative analysis, two of them [6, 47] were excluded for the meta-analysis because did not meet the inclusion criteria. Whereas, Aroca et al. [6] provided the results of CRC at the level of the intervened subjects, Gupta et al. [47] informed of the obtained coverage at 9 months which was a time-point not included in the pre-established analysis groups. The meta-analysis (Fig. 4) showed a high heterogeneity between studies which prevented obtaining significance when comparing pre-twenty-first century techniques versus twenty-first century techniques. Even so, a certain trend could be observed in the cumulative meta-analysis (Fig. 5), with the results of CRC tending to improve in the most current studies with a 12-month follow-up, whereas in those with a follow-up beyond 12 months, the opposite trend was seen.

## Discussion

The present systematic review considered all types of studies which focused on Miller class III [4] or RT2 [5] GRs, due to the lack of evidence regarding the CRC of these challenging GRs. Until now, only nine [31, 36, 38, 39, 42, 50, 51, 53, 68] out of the 37 included studies showed a follow-up longer than 12 months, one of them being a randomized clinical trial [42]. Furthermore, only two studies [38, 51] had a long-term follow-up of five and twenty years, respectively.

It was decided to divide the surgical techniques using the twenty-first century as a threshold, because it is considered that, from that moment on, new techniques or modifications of the previous original techniques had been described, which were headed towards minimally invasive surgery, in an attempt to overcome the difficulties, the former presented.

Considering globally all the selected studies in this systematic review, CRC is possible, but the percentage of the CRC seems to decrease exponentially with a longer follow-up time (six months: 54.88%; 12 months: 42.07% and > 12 months: 18.23%). In this way, the study by Pini-Prato et al. [51] which had the longest follow-up, was the one showing no stability of the CRC in the 17 recessions after 20 years of follow-up. When considering both groups (pre-twenty-first century [17–27] and twenty-first century [6, 28–42] techniques) independently, both of them showed CRC in half of the treated recessions (pre-twenty-first: 57.60% vs. 21st: 51.11%) at six months. The CRC decreased markedly for the twenty-first century [6, 28–42] techniques at 12 months (pre-twenty-first: 63.82% vs. 21st: 32.87%). Nevertheless, beyond the 12-month follow-up, the tendency of the CRC was inverted (pre-twenty-first: 5.26% vs. 21st: 19.65%). This could be due to the higher number of recessions treated with the more current techniques at 12 months (pre-twenty-first: n = 152 vs. 21st: n = 359), and beyond 12 months (pre-twenty-first: n = 19 vs. 21st: n = 173). In fact, the number of recessions treated with the more modern techniques were more than the double at 12 months and more than nine times beyond 12 months; it might explain the large differences in CRC. In addition, other factors related to the defect, the patient and the surgical characteristics may have influenced the CRC obtained.

At recession level, there were other parameters that may have influenced the achievement of the desired CRC, such as interproximal soft tissue integrity [67, 69, 70], avascular root surface areas (AERSA) [5, 63] size of the recession (baseline depth and width) [27, 56, 71], periodontal biotype [69], which directly influences the flap thickness [72] and, finally, tooth and location. Thus, it was more difficult to achieve CRC in GRs located in the mandible [73], as well as in molars and premolars [74, 75]

It must be noted that an attempt was made to elucidate what baseline parameters may favor achieving CRC in recessions in which, until now, only a partial coverage could be achieved. Considering the characteristics of the recessions, in the consensus report of the 10th European Workshop on Periodontology, it was stated that interproximal attachment loss would not necessarily be a prognostic limitation to obtain a successful root coverage [4, 76]. As a matter of fact, a similar behavior has been observed in GRs with no interproximal attachment loss and in those with a loss of interproximal attachment ≤ 3 mm [7]. Hence, this should not be the only parameter to consider when trying to predict successful coverage [8].

On the other hand, there would also be patient-related factors, such as bad oral hygiene, poor general health or smoking, which have been associated with negative results when performing any periodontal surgery [54, 77]. Therefore, the same might be expected for mucogingival surgery, but to our knowledge, there is no clinical study that has assessed the influence of delayed healing associated with certain determined systemic diseases on root coverage. In fact, the majority of patients in these studies usually showed good oral hygiene and general health, and were non-smokers, so these results should initially be extrapolated only to this kind of patients. Furthermore, while the analysis at the patient level may be more clinically relevant as it might allow assessing the results on each surgery [6], it would also be interesting to analyze results at recession level in order to know the amount of coverage can be achieved with each technique and to enable comparison between studies.

In this review, we have focused on surgical techniques, in which many variables might also influence a successful coverage, such as flap tension [78], position of the gingival margin coronally to the CEJ after suturing [79], root surface treatment [80], the surgeon´s skills with root coverage improving along with surgical experience [81, 82], which would also be reflected in the so-called “center effect” [76, 83], the type of graft, and finally, the thickness of the subepithelial connective tissue graft. Although connective tissue graft is still considered as the gold standard [8], multiple materials are being developed in an attempt to avoid a second surgical site while achieving the same results. Concerning thickness of the autogenous graft, some authors advocated a graft thicker than 2 mm [50, 67] for better results of CRC. On the other hand, other authors suggested that the thicker the graft, the greater the difficulties it will have for its vascularization and the worse the aesthetic results will be [84].

When the cumulative meta-analysis was performed, better results of CRC were observed in the more recent studies with a 12-month follow-up, regardless of the technique used. However, the opposite was seen when the follow-up was longer than 12 months. Improved knowledge, materials and techniques, such as microsurgery [85] may provide better results in achieving CRC in the short term, but it seems that the ageing of tissues, in particular changes in the thickness of the periodontium, could generate a biological remodeling due to long-term environmental influence [51], unlike what would be expected at around 12 months because of the “creeping attachment” [86] of the periodontal tissues. The difficulty lies in knowing at what point this initial maturation, which favors root coverage, begins to age and to be detrimental to the results obtained.

In terms of the limitations of the present review, the scarce evidence available for the treatment of Class III [4] or RT2 [5] GRs should be highlighted, as opposed to the existing evidence for Class I/II [4] or RT1 [5] recessions. In addition, the vast majority of the studies were case reports or case series, so the results should be viewed with caution since there was a high risk of bias, in many cases due to the lack of a masked operator and a blind examiner. It should also be considered that with the available measuring instruments, only linear measurements could be carried out, especially when trying to assess the extent of the avascular area of a recession. Thus, advances in digital [69] and three-dimensional techniques could suppose a great benefit.

Also, most of the studies had a short follow-up, which was insufficient to elucidate what will happen with CRC in the long-term. Matter [86] postulated that “creeping attachment” might happen up to one year after the surgical intervention, which would improve the clinical results in gingival recessions. As this has been extrapolated to all mucogingival techniques when treating any type of recession, including Miller class III [4] or RT2 [5] GRs, the percentage of CRC registered after a year (42.07%) should not be lower than that obtained at six months (54.88%), in contrast to what was noted in this review. Matter [86] pointed that the stability of the coverage was maintained after five years of follow-up. Instead, Pini-Prato et al. [51], stated that in Miller class III [4] or RT2 [5] GRs the stability of the CRC was not possible after a follow-up of 20 years. It has been suggested that different factors, such as the presence of an attached keratinized tissue band smaller than 2 mm, the absence of interdental periodontal tissue or ageing, might be responsible for this fact [51]. Therefore, longer follow-ups in the same type of recessions, assessing the healing and stability of the tissues in the long term, could help to clarify this critical issue to clinicians, together with collecting more clinical variables, that, as it has been observed, could influence the results of root coverage.

Although the surgical technique and the type of recession influence the results of CRC, there are multiple other variables that are known to condition this outcome and that need to be investigated independently in order to know their correlation with CRC, such as the periodontal phenotype, absence of keratinized gingiva, tooth location, dimension and position (tooth extrusion, rotation or vestibular displacement) and the presence of frenula or shallow vestibular depth.

## Conclusions

Within the limits of this review, it can be stated that it might be possible to achieve CRC, regardless of which technique is used, but its stability is not predictable. Nevertheless, there are many low-quality studies for the treatment of Miller class III or RT2 recessions, with short follow-up times. Hence, more randomized clinical trials are needed, with longer follow-ups and with several visits to assess the outcomes of the root coverage and the effectiveness of the surgical procedures, in order to develop more predictable techniques and to confirm the stability of the results achieved in Miller class III or RT2 recessions. Moreover, studies assessing the patient’s satisfaction in relation with the clinical outcomes (mean root coverage, the state of the surrounding tissues), might be necessary in order to establish success criteria in the treatment of these recessions.

## Supplementary Information

**Additional file 1**. Excluded studies and reasons for their exclusion. The additional file 1 shows a table including all the excluded studies and the reasons of their exclusion. All the references of these excluded studies are inside the additional file 1.

## Data Availability

All data generated or analysed during this study are included in this published article [and its supplementary information files].

## Abbreviations

GRs: Gingival recessions
RC: Root coverage
CRC: Complete root coverage
PRISMA: Preferred Reporting Items for Systematic Review and Meta-Analysis
PROSPERO: International Prospective Register of Ongoing Systematic Reviews
GRADE: Grading of Recommendations. Assessment, Development and Evaluation
AF1: Additional File 1
TT: Tunnel technique
MCAT: Modified coronally advanced tunnel
VISTA: Vestibular incision subperiosteal tunnel access
CAF: Coronally advanced flap
PST: Pinhole surgical technique
LCT: Laterally closed tunnel
BFP: Bridge flap procedure
FGG: Free gingival graft
PCTG: Periosteal connective tissue grafting
MCAF: Modified coronally advanced flap
GUT: Gingival unit transfer
ROT: Rotated Technique
DPG: Double pedicle graft
LMCAF: Laterally moved coronally advanced flap
MLPF: Modified laterally positioned flap
LPF: Laterally positioned flap
2SSA: Two-Step Surgical Approach
AERSA: Avascular root surface areas
CEJ: Cementum enamel junction
S: Single
RC: Recession
M: Multiple
Mx: Maxilla
Md: Mandible
NR: No reported
ADM: Acelullar dermal matrix
XCM: Xenogenic collagen matrix
SCTG: Subepithelial connective tissue graft
BB: Bioabsorbable barrier
EMD: Enamel matrix derivative
PRF: Platelet-rich fibrin
PDGF: Platelet derived growth factor
Mo: Months
PD: Probing depth
CAL: Clinical attachment level
KTW: Keratinized tissue width
