# Correction to: Complete root coverage in the treatment of Miller class III or RT2 gingival recessions: a systematic review and meta-analysis

**DOI:** 10.1186/s12903-021-01537-9

**Published:** 2021-04-26

**Authors:** Aitziber Fernández-Jiménez, Ana-María García-De-La-Fuente, Ruth Estefanía-Fresco, Xabier Marichalar-Mendia, José-Manuel Aguirre-Urizar, Luis-Antonio Aguirre-Zorzano

**Affiliations:** 1 Department of Stomatology II, University of the Basque Country (UPV/EHU), UPV/EHU. Barrio Sarriena S/N, 48940 Leioa, Biscay Spain; 2 Department of Nursing I, University of the Basque Country (UPV/EHU), Barrio Sarriena S/N, 48940 Leioa, Biscay Spain

## Correction to: BMC Oral Health. 10.1186/s12903-021-01494-3

Following publication of the original article [1], the authors identified errors in Figs. 4 and 5. The correct figures are given below.

**Fig. 4:**
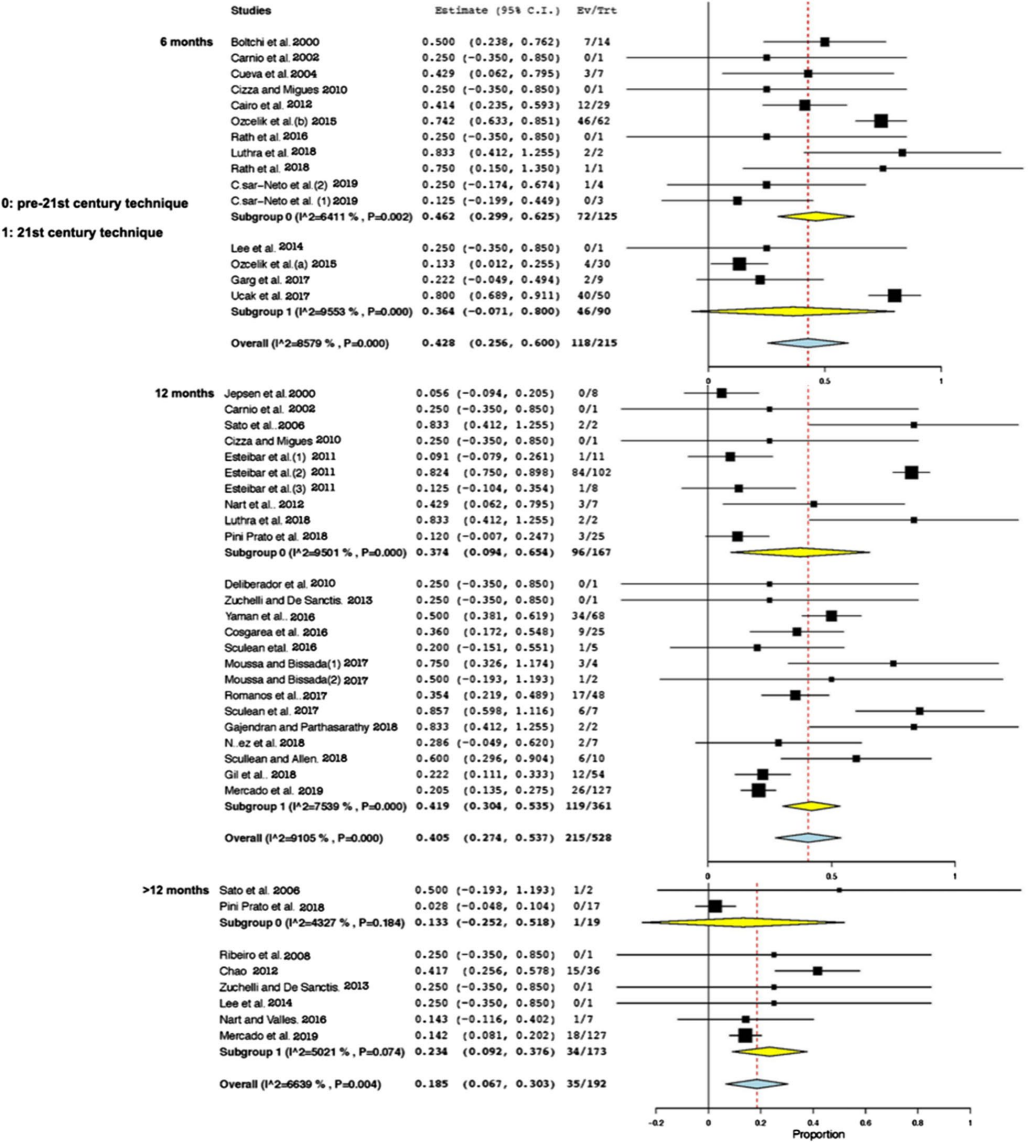
Random-effects model analyses comparing pre-twenty-first century and twenty-first century techniques

**Fig. 5:**
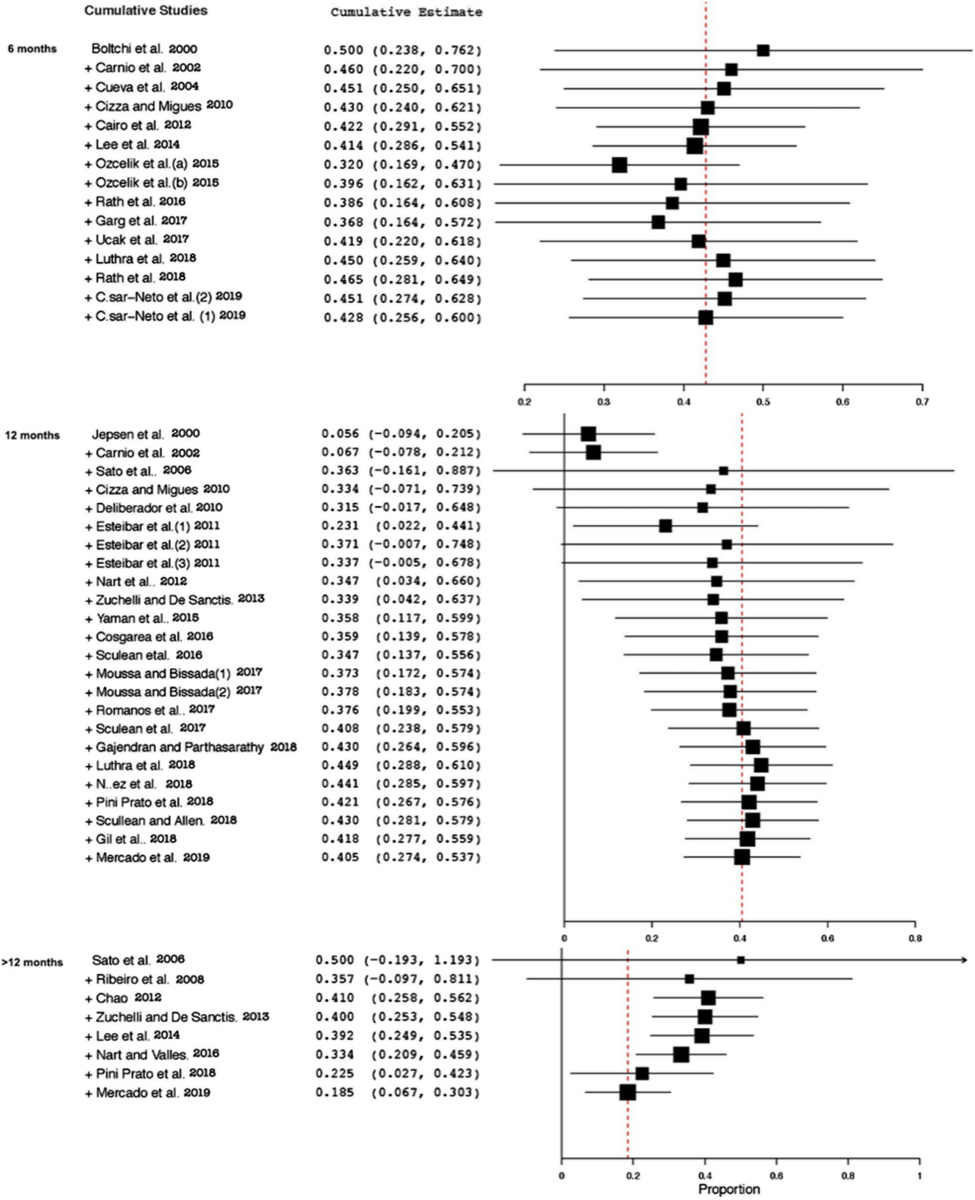
Cumulative meta-analysis for all studies

The original article [1] has been corrected.
