# Supplementary material for: Complete root coverage in the treatment of Miller class III or RT2 gingival recessions: a systematic review and meta-analysis
Source: BMC Oral Health. 2021 Mar 22;21:145. doi: 10.1186/s12903-021-01494-3 (PMC7986294; doi:10.1186/s12903-021-01494-3)
Supplement: Supplementary file 1 — Additional file 1. Excluded studies and reasons for their exclusion. The additional file 1 shows a table including all the excluded studies and the reasons of their exclusion. All the references of these excluded studies are inside the additional file 1. [file 12903_2021_1494_MOESM1_ESM.pdf]

Table S1. Excluded studies from the systematic review with reasons

Exclusion criteria	Excluded studies (n=37)	
**No Class III or RT2**	4	Agarwal et al. 2014, Bonacci 2011, Purushotham et al. 2016, Wilcko et al. 2005
**No complete root coverage for Class III or RT2**	16	Barker et al. 2010, Carney et al. 2012, Cheng et al. 2015, Deepa and Kumar 2018, Henriques et al. 2010, Jovicic et al. 2008, Kerner et al. 2008, Lee et al. 2002, Müller et al. 2000, Pini-Prato et al. 2010, 2015, Remya et al. 2008, Schlee et al. 2014, Shin et al. 2007, Stähli et al. 2019, Zucchelli et al. 2010
**No mucogingival resolution for Class III or RT2**	2	De Molon et al. 2012, Zucchelli et al. 2012
**Follow-up less than 6 months**	6	Comuzzi et al. 2014, Ioannou et al. 2016, Kolhatkar et al. 2010, Mahn 2015, McLeod et al. 2009, Park 2010
**Incomplete data**	9	Allen and Winter 2011, Blanes and Allen 1999, Ercan et al. 2016, Geisinger et al. 2016, Ito et al. 2000, Kumari et al. 2010, Ogata et al. 2017, Panda et al. 2016, Ross and Ross 2019

Agarwal C, Gayathri GV, Mehta DS. An innovative technique for root coverage using pedicled buccal fat pad. Contemp Clin Dent. 2014;5:386-388.

Allen EP, Winter RR. Interdisciplinary treatment of cervical lesions. Compend Contin Educ Dent. 2011;32:16-20.

Barker TS, Cueva MA, Rivera-Hidalgo F, et al. A comparative study of root coverage using two different acellular dermal matrix products. J Periodontol. 2010;81:1596-1603.

Blanes RJ, Allen EP. The bilateral pedicle flap-tunnel technique: a new approach to cover connective tissue grafts. Int J Periodontics Restorative Dent.1999;19:471-479.

Bonacci FJ. Hard and soft tissue augmentation in a postorthodontic patient: a case report. Int J Periodontics Restorative Dent. 2011;31:19-27.

Carney CM, Rossmann JA, Kerns DG, et al. A comparative study of root defect coverage using an acellular dermal matrix with and without a recombinant human platelet-derived growth factor. J Periodontol. 2012;83:893-901.

Cheng GL, Fu E, Tu YK, et al. Root coverage by coronally advanced flap with connective tissue graft and/or enamel matrix derivative: a meta-analysis. J Periodontal Res. 2015;50:220-230.

Comuzzi L, Mazzocco F, Stefani R, et al. Human histologic evaluation of root coverage obtained with connective tissue graft over a compomer restoration. Int J Periodontics Restorative Dent. 2014;34:39-45.

De Molon RS, De Avila ÉD, De Souza JAC, Nogueira AVB, Cirelli CC, Cirelli JA. Combination of orthodontic movement and periodontal therapy for full root coverage in a miller class III recession: A case report with 12 years of follow-up. Braz Dent J. 2012;23:758-763.

Deepa D, Arun Kumar KV. Clinical evaluation of Class II and Class III gingival recession defects of maxillary posterior teeth treated with pedicled buccal fat pad: A pilot study. Dent Res J (Isfahan) 2018;15:11-16

Ercan E, Candirli C, Uysal C, Uzun BC, Yenilmez E. Treatment of Severe Gingival Recession Using Pedicled Buccal Fat Pad: Histological and Clinical Findings. Clin Exp Health Sci. 2016;6:191-4.

Geisinger, M. L., Trammell, K., Holmes, C. M., Kaur, M., & Geurs, N. C. Does adjunctive use of growth factors improve clinical outcomes of soft tissue grafting at Miller Class III recession defects? A review of current evidence. Clin adv periodontics 2016;6: 99-103.

Henriques PS, Pelegrine AA, Nogueira AA, Borghi MM. Application of subepithelial connective tissue graft with or without enamel matrix derivative for root coverage: a split-mouth randomized study. J Oral Sci. 2010;52:463-471

Ioannou AL, Kotsakis GA, Kamintzi GI. Complete Coverage of a Class III (RT2) Gingival Recession With the Combination of a Free Subepithelial Connective Tissue Graft and a Laterally Positioned Flap. Clin Adv Periodontics. 2016;6:1-7.

Ito K, Owa M. Connective tissue grafting for root coverage in multiple Class III gingival recessions with enamel matrix derivative: a case report. Pract Periodontics Aesthet Dent. 2000;12:441-6; quiz 448.

Jovicic B, Lazic Z, Nedic M. Therapeutic efficacy of guided tissue regeneration and connective tissue autotransplants with periosteum in the management of gingival recession. Vojnosanitetski Pregled. 2008;65:758-762.

Kerner S, Borghetti A, Katsahian S, et al. A retrospective study of root coverage procedures using an image analysis system. J Clin Periodontol. 2008;35:346-355.

Kolhatkar S, Haque SA, Winkler JR, Bhola M. Root coverage in an HIV-positive individual: combined use of a lateral sliding flap and resin-modified glass ionomer for the management of an isolated severe recession defect. J Periodontol. 2010;81:632-640.

Kumari BN, Thiagarajan R, Narayanan V, Devadoss P, Mammen B, Emmadi P. A new technique for root coverage using buccal fat pad--a short case report. Quintessence Int. 2010;41:547-549.

Lee YM, Kim JY, Seol YJ, et al. A 3-year longitudinal evaluation of subpedicle free connective tissue graft for gingival recession coverage. J Periodontol. 2002;73:1412-1418.

Mahn DH. Minimizing shrinkage of interdental papilla height when treating multiple Miller Class III gingival recession defects. Compend Contin Educ Dent. 2015;36:275-276, 279-281.

McLeod DE, Reyes E, Branch-Mays G. Treatment of multiple areas of gingival recession using a simple harvesting technique for autogenous connective tissue graft. J Periodontol. 2009;80:1680-1687.

Müller HP, Stahl M, Eger T. Failure of root coverage of shallow gingival recessions employing GTR and a bioresorbable membrane. Int J Periodontics Restorative Dent. 2001;21:171-181.

Ogata Y, Bui M, Griffin TJ, Hur Y. Use of Allograft with Platelet Concentrate in the Treatment of Multiple Miller Class III Gingival Recession Defects: Report of Three Cases. Int J Periodontics Restorative Dent. 2017;37:339-344.

Panda S, Del Fabbro M, Satpathy A, Das AC. Pedicled buccal fat pad graft for root coverage in severe gingival recession defect. J Indian Soc Periodontol. 2016;20:216-219.

Park JB. A two-stage approach using an autogenous masticatory mucosal graft and an autogenous connective tissue graft to treat gingival recession: a case report. J Int Acad Periodontol. 2010;12:45-48.

Pini-Prato G, Magnani C, Zaheer F, Rotundo R, Buti J. Influence of inter-dental tissues and root surface condition on complete root coverage following treatment of gingival recessions: a 1-year retrospective study. J Clin Periodontol. 2015;42:567-74.

Pini-Prato GP, Cairo F, Nieri M, Franceschi D, Rotundo R, Cortellini P. Coronally advanced flap versus connective tissue graft in the treatment of multiple gingival recessions: a split-mouth study with a 5-year follow-up. J Clin Periodontol. 2010;37:644-650.

Purushotham S, Manjunath N, D'Souza ML, Shetty R. An interdisciplinary approach for the management of noncarious lesions. J Indian Soc Periodontol. 2016;20:211-215.

Remya V, Kishore Kumar K, Sudharsan S, Arun K. Free gingival graft in the treatment of class III gingival recession. IJDR. 2008;19:247-252.

Ross B, Ross SB. Cryopreserved Umbilical Cord Allograft for Root Coverage of Gingival Recession Defects: A Case Series. Int J Periodontics Restorative Dent. 2019;39:391-397.

Schlee M, Lex M, Rathe F, Kasaj A, Sader R. Treatment of multiple recessions by means of a collagen matrix: a case series. Int J Periodontics Restorative Dent. 2014;34:817-823.

Shin SH, Cueva MA, Kerns DG, Hallmon WW, Rivera-Hidalgo F, Nunn ME. A comparative study of root coverage using acellular dermal matrix with and without enamel matrix derivative. J Periodontol. 2007;78:411-421.

Stahli A, Imber JC, Raptis E, Salvi GE, Eick S, Sculean A. Effect of enamel matrix derivative on wound healing following gingival recession coverage using the modified coronally advanced tunnel and subepithelial connective tissue graft: a randomised, controlled, clinical study. Clin Oral Investig. 2020;24:1043-1051.

Wilcko MT, Wilcko WM, Murphy KG, et al. Full-thickness flap/subepithelial connective tissue grafting with intramarrow penetrations: three case reports of lingual root coverage. Int J Periodontics Restorative Dent. 2005;25:561-569.

Zucchelli G, Mele M, Stefanini M, et al. Predetermination of root coverage. J Periodontol. 2010;81:1019-1026.

Zucchelli G, Parenti SI, Ghigi G, Bonetti GA. Combined orthodontic - mucogingival treatment of a deep post-orthodontic gingival recession. Eur J Esthet Dent. 2012;7:266-280.
